# Fatal Metacestode Infection in Bornean Orangutan Caused by Unknown *Versteria* Species

**DOI:** 10.3201/eid2001.131191

**Published:** 2014-01

**Authors:** Tony L. Goldberg, Annette Gendron-Fitzpatrick, Kathleen M. Deering, Roberta S. Wallace, Victoria L. Clyde, Michael Lauck, Gail E. Rosen, Andrew J. Bennett, Ellis C. Greiner, David H. O’Connor

**Affiliations:** University of Wisconsin, Madison Wisconsin, USA (T.L. Goldberg, A. Gendron-Fitzpatrick, K.M. Deering, G.E. Rosen, A.J. Bennett, D.H. O’Connor);; Milwaukee County Zoo, Milwaukee, Wisconsin, USA (A. Gendron-Fitzpatrick, K.M. Deering, R.S. Wallace, V. L. Clyde);; University of Florida, Gainesville, Florida, USA (E.C. Greiner);; Wisconsin National Primate Research Center, Madison (T.L. Goldberg, D.H. O’Connor).

**Keywords:** Cestoda, Taeniidae, *Versteria*, metacestode, primate, orangutan, *Pongo pygmaeus*, deep sequencing, parasites

## Abstract

A captive juvenile Bornean orangutan (*Pongo pygmaeus*) died from an unknown disseminated parasitic infection. Deep sequencing of DNA from infected tissues, followed by gene-specific PCR and sequencing, revealed a divergent species within the newly proposed genus *Versteria* (Cestoda: Taeniidae). *Versteria* may represent a previously unrecognized risk to primate health.

We describe the identification of a previously genetically uncharacterized species within the newly proposed Taeniid (Cestoda) genus *Versteria* (*1*), which caused fatal metacestode infection in a captive juvenile Bornean orangutan (*Pongo pygmaeus*). The orangutan was born in Colorado, USA, on April 4, 2007, and, after maternal rejection, was transported to the Milwaukee County Zoo in Milwaukee, Wisconsin, USA, for adoption by a surrogate mother on February 7, 2008. On December 27, 2012, keepers noted that the orangutan was exhibiting loss of appetite and an intermittent, moist cough. The animal became increasingly lethargic and was found dead 2 days later.

## The Study

**Figure 1:**
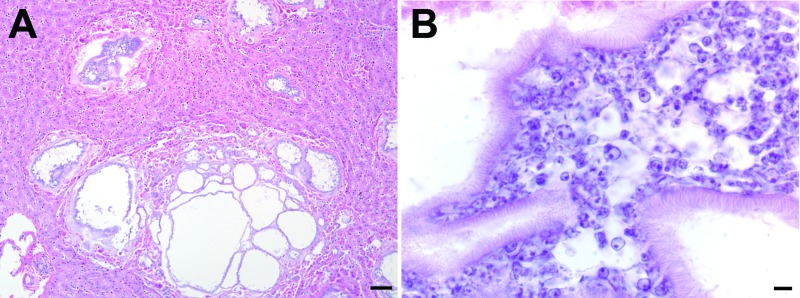
Microscopic images of liver sections from a Bornean orangutan fatally infected with *Versteria* metacestodes. Images of liver sections stained with hematoxylin and eosin (H&E) stain were captured at 10× magnification (A; scale bar = 30 μm) and 100× magnification; B; scale bar = 5 μm). Large numbers of parasite cells can be seen within well-defined cystic structures separated from the surrounding host tissue by clearly visible membranes.

Postmortem examination revealed diffuse hemorrhages in the lungs (which did not collapse), splenomegaly, a pale mottled liver, and thoracic and pericardial effusions. Diagnostic microbiologic examination of tracheal washes and lung tissue identified only common environmental bacteria, and tests for viruses and fecal examination for parasites were all negative. Histopathologic examination of the liver revealed cystic structures containing eukaryotic parasite cells between ≈4 and 5 μm in diameter (Figure 1). Similar cells were observed in the parenchyma and blood vessels of lung and spleen (not shown). On the basis of these results and clinical observations, the cause of death was determined to be acute respiratory distress due to disseminated infection with an unknown parasite.

Because attempts to identify the parasite by morphologic features were inconclusive, total DNA extracted from infected organs was subjected to deep sequencing to detect molecular sequences of pathogens. DNA was isolated from liver, lung, and spleen by using the QIAGEN DNeasy Blood and Tissue Kit (QIAGEN Inc., Valencia, CA, USA), followed by treatment with RNase (Epicenter Biotechnologies, Madison, WI, USA) to remove RNA. DNA libraries were then generated by using the Nextera DNA Sample Prep Kit (Illumina, San Diego, CA, USA) and sequenced on an Illumina MiSeq instrument as described (*2*). Resulting sequence data were analyzed by using CLC Genomics Workbench 5.5 (CLC bio, Aarhus, Denmark). Briefly, low quality (<q30) and short (<100-bp) sequences were removed, sequences were aligned against an orangutan (*P. abelii*) genome (*3*), and nonmapped sequences were subjected to de novo assembly.

Deep sequencing of total DNA from infected tissues resulted in ≈2,400,000 sequences after quality trimming. Subtractive mapping against the orangutan genome removed ≈97% of these sequences. De novo assembly of the remaining ≈50,000 sequences resulted in 293 contiguous sequences, 7 of which had high similarity to GenBank sequences corresponding to *Taenia* spp. (expected values <1 × 10^−18^). Subsequent mapping of nonhost sequences against the *T. solium* genome (*4*) resulted in 8,494 matches.

On the basis of deep-sequencing results, PCR primers were used to amplify 3 mitochondrial genes informative for resolving relationships within the Taeniidae (Table). For 12s ribosomal RNA (12s rRNA), primers CES12sF (5′-AGGGGATAGGACACAGTGCCAGC-3′) and CES12sR (5′-CGGTGTGTACMTGAGYTAAAC-3′) were modified from GenBank accession nos. KC344674–KC344701. For cytochrome c oxidase subunit I (*cox1*), published primers JB3 (5′-TTTTTTGGGCATCCTGAGGTTTAT-3′) and JB4.5 (5′-TAAAGAAAGAACATAATGAAAATG-3′) were used, and for NADH dehydrogenase subunit 1 (*nad1*), published primers JB11 (5′-AGATTCGTAAGGGGCCTAATA- 3′) and JB12 (5′-ACCACTAACTAATTCACTTTC-3′) were used (*5*). PCRs were conducted in 20-μL volumes with 1-μL DNA template by using the Phusion kit (New England Biolabs Inc., Ipswich, MA, USA), cycled as follows: 98°C, 30 s; 35 cycles of 94°C, 10 s, annealing, 30 s, 72°C, 30 s; and final extension at 72°C for 10 min (annealing temperatures for 12s rRNA, *cox1*, and *nad1* were 60°C, 50°C, and 55°C, respectively).

**Table:** GenBank accession numbers of taeniid DNA sequences used in phylogenetic analyses

Taxon	Origin	GenBank accession nos.*		
		12s rRNA	*cox1*	*nad1*
*Echinococcus*				
* E. canadensis*	Kazakhstan	NC_011121	NC_011121	NC_011121
* E. equinus*	United Kingdom	AF346403	AF346403	AF346403
* E. felidis*	Uganda	AB732958	AB732958	AB732958
* E. granulosus*	United Kingdom	NC_008075	NC_008075	NC_008075
* E. multilocularis*	Japan	NC_000928	NC_000928	NC_000928
* E. oligarthrus*	Panama	NC_009461	NC_009461	NC_009461
* E. ortleppi*	Argentina	NC_011122	NC_011122	NC_011122
* E. shiquicus*	Tibet	NC_009460	NC_009460	NC_009460
* E. vogeli*	Colombia	NC_009462	NC_009462	NC_009462
*Hydatigera*				
* H. krepkogorski*	China		AB731762	AB731762
* H. parva*	Spain		AB731760	AB731760
*H. taeniaeformis* (A)	China	NC_014768	NC_014768	NC_014768
*H. taeniaeformis* (B)	Finland		AB731761	AB731761
*Taenia*				
* T. arctos*	Finland		GU252130	GU252132
* T. asiatica*	Korea	NC_004826	NC_004826	NC_004826
* T. crassiceps*	Canada	NC_002547	NC_002547	NC_002547
* T. hydatigena*	China	NC_012896	NC_012896	NC_012896
* T. krabbei*	Norway		EU544578	EU544631
* T. laticollis*	Finland		AB731727	AB731727
* T. madoquae*	Kenya		AB731726	AB731726
* T. martis*	Croatia	NC_020153	NC_020153	NC_020153
* T. multiceps*	China	NC_012894	NC_012894	NC_012894
* T. multiceps gaigeri*	Iran		HM101469	HM101470
* T. omissa*	Canada		JX860631	JX860632
* T. ovis*	New Zealand	AB731675	AB731675	AB731675
* T. pisiformis*	China	NC_013844	NC_013844	NC_013844
* T. polyacantha arctica*	Greenland		EU544594	EU544646
* T. polyacantha polyacantha*	Denmark		EU544583	EU544636
*T. regis* (A)	Kenya		AM503328	AM503346
*T. regis* (B)	Kenya		AM503329	AM503347
* T. saginata*	Africa	NC_009938	NC_009938	NC_009938
*T. serialis* (A)	Kenya		AM503319	AM503336,
*T. serialis* (B)	Kenya		AM503322	AM503339
* T. solium*	China	NC_004022	NC_004022	NC_004022
*T.* sp. AL-2012	Finland		JX860629	JX860630
* T. twitchelli*	Russia		AB731759	AB731759
*Versteria*				
*V. mustelae* (A)	Finland		EU544567	EU544620
*V. mustelae* (B)	Russia		EU544571	EU544624
*V*. sp.†	United States	KF303339	KF303340	KF303341

*Multiple sequences were chosen to capture the maximum extent of intraspecific genetic divergence within highly diverse taxa (variants arbitrarily labeled A or B). †Sequences generated in this study.

Amplicons underwent electrophoresis on 1% agarose gels stained with ethidium bromide, were purified with the Zymoclean Gel DNA Recovery Kit (Zymo Research, Irvine, CA, USA), and were Sanger-sequenced in both directions by using PCR primers on ABI 3730xl DNA Analyzers (Applied Biosystems, Carlsbad, CA, USA) at the University of Wisconsin Biotechnology Center. Sequence chromatograms were edited and assembled by using Sequencher version 4.9 (Gene Codes Corporation, Ann Arbor, MI, USA). Sequences were aligned with homologous sequences from all taeniid species in GenBank as of April 7, 2013 (Table). To construct phylogenetic trees, we used the maximum-likelihood method in MEGA5.2 software (*6*).

**Figure 2:**
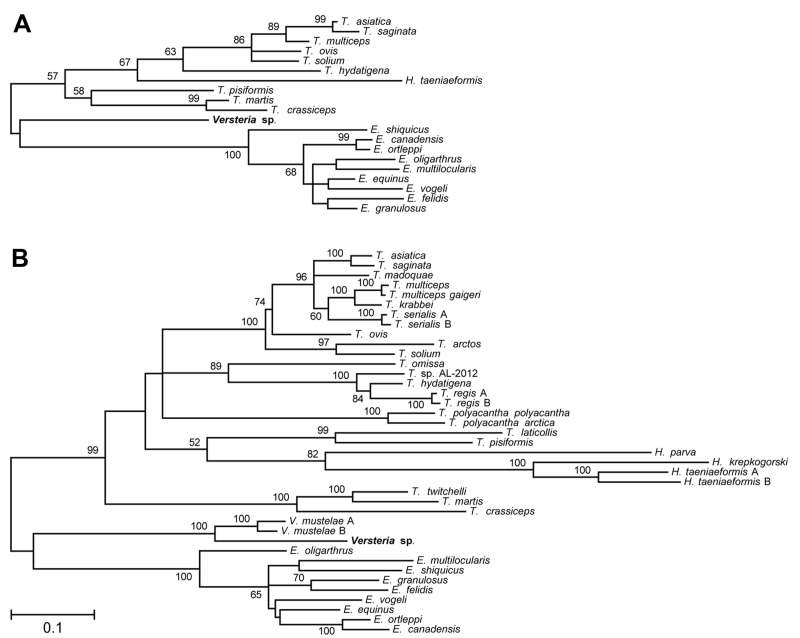
Phylogenetic trees of the Taeniidae, including newly generated sequences derived from tissues of a fatally infected Bornean orangutan. Trees were constructed from DNA sequence alignments of 12s rRNA (A) and concatenated *cox1*/*nad1* (B) sequences from the orangutan (*Versteria* sp.; bold; accession nos. KF303339–303341) and representative *Echinococcus*, *Hydatigera*, *Taenia,* and *Versteria* sequences from GenBank (see Table). The maximum likelihood method was used, with the likeliest model of molecular evolution chosen for both datasets by using MEGA5.2 software (*6*). Models of molecular evolution and tree likelihood values are HKY+G, -lnL = 2279.42 for 12s rRNA, and GTR+G+I, -lnL = 11582.71 for *cox1*/nad1. Numbers next to branches indicate bootstrap values (%), estimated from 1,000 resamplings of the data (only bootstrap values ≥50% are shown). Scale bar indicates nucleotide substitutions per site.

Figure 2 shows phylogenetic trees of newly generated 12s rRNA (panel A) and concatenated *cox1*/*nad1* (panel B) sequences and representative taeniid sequences. The trees closely agree with recently published taeniid phylogenies (*1*). Concatenated *cox1*/*nad1* sequences from the orangutan cluster with *V. mustelae* (formerly, *T. mustelae*) with 100% bootstrap support, placing the organism within the newly proposed genus *Versteria* (*1*) with confidence. However, the new *cox1* and *nad1* sequences are ≈12% different from those of published *V. mustelae* sequences. This degree of divergence is equal to or greater than that separating established *Echinococcus* and *Taenia* spp. (*7*) (Figure 2).

Members of the newly proposed genus *Versteria* have morphologic features that distinguish them from members of the other taeniid genera, such as miniature rostellar hooks, small scolex, rostellum, and suckers; a short strobili; and a small number of testes (*1*). However, no such distinguishing morphologic features could be identified by microscopy in the case described here. *V. mustelae* tapeworms infect multiple small animal intermediate host species and have been found in the upper midwestern United States in a hunter-killed fox squirrel (*Sciurus niger rufeventer*) with hepatic cysts (*8*). The definitive hosts of *V. mustelae* tapeworms are small carnivores of the family Mustelidae, such as weasels and martens (*9*). The genus *Versteria* also contains *V. brachyacantha* (*10*) tapeworms, which infect the African striped weasel (*Poecilogale albinucha*), but sequences of this species are not represented in GenBank. North American *V. mustelae* tapeworms are capable of asexual multiplication in the intermediate host (*11*); however, sequence data are only available for Eurasian specimens (*7*). The parasite described herein could thus represent a novel species or a previously genetically uncharacterized North American *V. mustelae* variant.

## Conclusions

This study illustrates the utility of deep sequencing for diagnosing and characterizing enigmatic parasites. Similar methods have aided in the discovery of RNA viruses (*12*), but their application to eukaryotic pathogens has lagged, presumably because of technical challenges associated with distinguishing host from parasite DNA. In this light, it is noteworthy that our efforts were greatly facilitated by the availability of an orangutan genome against which to perform in silico subtractive mapping (*3*). As more host genomes become available, and as costs of equipment, reagents, and bioinformatics software decline, such methods promise to enter the diagnostic mainstream, as a complement to traditional morphologic and molecular approaches.

Encysted taeniid metacestodes can remain dormant for years before asexual multiplication (*13*); thus, this animal could have become infected at virtually any point in its life. Rapid progression to fatal disease could indicate an underlying condition, such as immune deficiency. Alternatively, this particular *Versteria* species may be inherently virulent. 

Regarding source of infection, orangutans engage in geophagy (*14*), a behavior that this animal frequently practiced, suggesting that the infectious agent could have been obtained from contaminated soil. However, other sources (e.g., food, water, fomites) cannot be excluded. Infectious eggs could have entered the orangutan’s environment through direct deposition by a definitive host or through complex pathways of environmental transport. To date, no other animals in the zoologic collections in Colorado or Wisconsin, where the orangutan was housed, have experienced similar disease, nor have similar infections been reported in persons, to our knowledge.

In any case, this animal’s rapid and severe disease progression raises concerns about the health of captive apes in similar settings. Moreover, the close evolutionary relationship between orangutans and humans (*3*) raises concerns about the parasite’s zoonotic potential. 
